# Public Health Student's Attitudes Toward Research

**DOI:** 10.3389/fpubh.2021.801249

**Published:** 2022-02-02

**Authors:** Benjamin R. Chand, Crystal Eio, Annastasia Alysandratos, Jake Thompson, Tam Ha

**Affiliations:** School of Health and Society, University of Wollongong, Wollongong, NSW, Australia

**Keywords:** master public health, research intention, research motivations, student research, postgraduate research, public health research, public health students, research attitudes

## Abstract

Research is able to improve the lives of big populations by investigating effective interventions and then implementing those through public health policies. Whilst research on the inclination of Medical or Science undergraduate and postgraduate students has been conducted, little is known about what students pursuing a Masters degree in Public Health perceive the purpose of research to be. Their perceptions and inclinations will shape their research pursuits and career directions, which impacts the health outcomes of the community. Our findings suggest MPH students see improving the lives of the community as the most important purpose of research. Student's had more inclination to pursue research when influenced by a mentor however, many students still claimed that they either lacked confidence and skills in completing research or had no intention of pursuing research beyond their degrees, which suggests the need for curriculum adjustments.

## Introduction

The field of public health can be credited with vast improvements in health and well-being by investigating effective interventions and then implementing those through public health policies. Public health research is critical to informing the evidence-based policies that will enable continued population health improvements and reductions in global health inequalities (1). It is crucial to understand the motivations of today's public health students as they will shape the research and policy decisions that determine future population health outcomes. Whilst research on the perceptions and motivations of medical students has been conducted, little is known about what public health students understand the purpose of research to be.

Studies examining the motivations of medical students have revealed the primary motivations for undertaking research were career advancement and to publish research; suggesting individualistic motivations (2–8). Zweigenthal et al. examined the motivations of Master of Public Health (MPH) students in South Africa showing that the primary motivations of physicians undertaking an MPH were to improve career prospects and to facilitate a transition to a career in research (8). Barriers to participation in research among medical students include a lack of mentors and a lack of research training (2–6, 9–14).

Despite recognition of the importance of research and positive perceptions of research among medical students, attracting physicians to research remains a challenge (2, 9, 15, 16). There is limited research exploring the perception of research among public health researchers. The COVID-19 pandemic has highlighted the importance of a highly qualified public health workforce, prompting calls for an increase in public health research and training programs (17–19).

To the best of our knowledge, no research has been identified exploring public health students' perceptions of research in an Australian context at an undergraduate or postgraduate level. This study aims to identify postgraduate public health students' perceptions, inclinations and barriers to research. The findings of this study will inform improvements in public health curricula.

## Methods

### Study Design and Location

A cross-sectional survey of postgraduate students enrolled in the Masters of Public Health (MPH) program at the University of Wollongong was conducted in the Autumn session of 2018. The MPH program consists of a 1.5-year program or a 2-year “Advanced” course. The course consists of a combination of coursework covering subjects such as health promotion, social determinants of health, health policy, epidemiology and health research methodologies finishing with a capstone Independent Study research elective. Students can choose to specialize in health promotion, public health nutrition, work health and safety, health informatics or public health research. All students enrolled in the MPH and MPH Advanced course in Autumn session 2018 were eligible for inclusion. Approval to conduct the study was obtained from the University of Wollongong Human Research Ethics Committee (ethics approval number 2017/400) prior to data collection.

### Questionnaire

The questionnaire consisted of three sections; demographic information, research inclination and perceptions of research purpose ranked on a scale from most important to least important. The survey questions were developed based on a review of the literature examining the perceptions and motivations of research among medical students (2, 4, 6, 12, 15, 20–22). Factors associated with research motivations, facilitators and barriers among medical students were used to develop items measuring research inclination in MPH students. Items examining the purpose of research were also drawn from the literature examining the research motivations among medical students and adapted to the aims of this study. An open-ended question was included to allow participants to explain why they had chosen their primary purpose of research. Survey questions were piloted for clarity with a small group of students. Minor amendments to the wording of survey questions were made.

### Data Collection

All students enrolled in the MPH program were invited to participate in the study by email sent by the Academic Program Director. Students were informed the survey was voluntary and advised of the purpose of the study to ensure they could make an informed choice to participate in the study. The data were collected online through a survey questionnaire platform, esurv.org. Two reminder emails were sent to students prior to the survey link terminating. Completion of the survey online constituted consent to participate in the study.

### Statistical Analysis

Data were analyzed using IBM SPSS Statistics version 23 (23). Categorical data were analyzed using chi-squared tests whilst continuous data were analyzed using *t*-tests and ANOVA. Several responses were merged to increase answer count and significance. Responses “yes” and “if yes” for the subject and semester were merged for the question “undertaken previous research subjects besides the Independent Study subject as part of the Master of Public Health subject.” The responses “completed” and “undertaking” were merged for the Independent Study question. For the question “do you have any interest in pursuing a research project outside of the MPH curriculum?,” the responses for “no” and “not sure” were merged for questions about confidence in doing research and interpreting research articles.

Age was found not to be normally distributed for both “not taking Independent Study” and “taking/completed Independent Study” thus, non-parametric statistics were used. Mann-Whitney U-test was run in place of the Independent Samples *t*-test to determine significance. Data were shown to be not normally distributed for both “to improve the lives of the community” and “other,” therefore, a Mann-Whitney U-test was run in place of the Independent Samples *t*-test.

## Results

Forty-five students enrolled in the MPH program participated in the study resulting in a response rate of 29%. Participants were 58% female and 62% were international students. The mean age of participants was 29.3 years (sd = 5.8, range = 24). Participant demographics are shown in Table 1. The majority of students (51%) were enrolled in their third or fourth semester out of the four-semester degree.

**Table 1:** Demographic characteristics of participants.

**Demographic Characteristic**	**Total (*n* = 45)**
**Gender (** ***n*** **, %)**	
Female	26 (58%)
Male	19 (42%)
**Age (mean, standard deviation)**	29.3 (5.8)
**Residential Status (** ***n*** **, %)**	
Domestic	17(38%)
International	28 (62%)
**Semester of study (** ***n*** **, %)**	
First	15 (32%)
Second	5 (11%)
Third	21 (45%)
Fourth	3 (6%)

Perceptions of research were ranked on a scale from most important (1) to least important (11) as shown in Table 2. “Improving the lives of the community” was ranked as most important by the largest number of students (31%) and also had the highest median response (median = 3). Improving research skills was ranked as most important by 29% of students and received the equal highest median response for research importance (median = 3). Progressing an academic career and finding a solution to a problem both received a median score of 4. The lowest median responses for importance were improving the rank of the University (median = 10), fame and recognition (median = 9), and obtaining a research grant (median = 9). Having a mentor had a significant impact on whether or not students ranked the option of improving the lives of the community as their first option (*p* = 0.010).

**Table 2:** Student's perceptions of the purpose of research.

	**Ranking from most important (1) to least important (11)**										
**Research purpose**	**1**	**2**	**3**	**4**	**5**	**6**	**7**	**8**	**9**	**10**	**11**
Pass the subject	12%	6%	2%	2%	18%	8%	23%	8%	8%	2%	10%
Publish a research paper	0%	16%	8%	16%	8%	14%	14%	16%	6%	0%	0%
Fame and recognition	2%	0%	4%	2%	2%	6%	14%	16%	14%	16%	23%
Progress academic career	14%	18%	14%	16%	16%	12%	2%	4%	0%	2%	0%
Achieve a higher salary	2%	6%	6%	8%	4%	6%	10%	8%	18%	14%	16%
Improve the lives of the community	31%	18%	14%	14%	10%	6%	2%	0%	4%	0%	0%
Find solutions to a problem	4%	29%	12%	14%	10%	10%	12%	6%	2%	0%	0%
Provide data to frame policy	2%	4%	8%	12%	14%	18%	12%	8%	6%	8%	6%
Improve research skills	29%	2%	27%	12%	12%	10%	0%	2%	4%	2%	0%
Obtain a research grant	2%	0%	2%	2%	4%	6%	8%	18%	27%	20%	10%
Improve ranking of the university	2%	0%	2%	0%	0%	2%	2%	12%	10%	35%	35%

Participants were also asked to explain why they chose their first option in free-text responses. Two themes were identified in the free-text responses; “improve the lives of the community” and “improve research skills.” Responses mirrored the findings of the quantitative analysis indicating altruistic motivations for undertaking research. A selection of free text responses to this question are shown in Table 3.

**Table 3:** Why students chose their first perception of the purpose of research.

**Free text responses**
“*I am a student and still in the learning phase. The first target of mine is to improve my research skills.”*
“*Improving the lives of the society is detrimental in the world. We need the generations to come live healthier and improve on their life expectancy. As a world, the problems we are facing are researched and great recommendations given but implementation and commitment by governments and other institutions fail the good work done. What policies do we need to discuss that support for this concerns in our society and who needs to be accountable to ensure this works for the best of the society we are living in.”*
“*I have learnt many new subjects and explored my knowledge in public health and with the knowledge I acquired in the academics I want to apply them back in my home country to improve communities and societies better.”*
“*The reasoning for this is because my main goal is to help the community and give back and improve the quality of health for the future.”*
“*It would be a satisfying feeling knowing in the near future I could use these skills to do something for our society and improve certain outcomes.”*
“*In the university, it is important to pass the subject.”*
“*After starting to study MPH course I have found passion in Health Policy and Research. Because of it, I wish to pursue a career in Research and Health Policy Development career.”*
“*Improve lives of the community – being a public health professional finding out the underlying cause behind the causation of disease and mortality and improve the lives of the community.”*
“*My main goal after my degree is finished is making a difference to disadvantaged populations.”*

Interest in conducting research after graduation (*n* = 34, 76%) and outside of the MPH program (*n* = 34, 78%) was high, however, fewer students (*n* = 17, 38%) expressed interest in pursuing a career in research. Only 29% (*n* = 13) of students were completing or had completed an Independent Study. The majority of students undertaking an Independent Study were female (79%) and domestic students (57%). Students undertaking an Independent Study were more likely to have a mentor (*p* = 0.004) and more likely to have completed other research subjects (*p* = 0.032). There was no evidence of a difference in interest in pursuing a research career (*p* = 0.637) or confidence in doing research (*p* = 0.428) between students undertaking an Independent Study and those not undertaking an Independent Study. A comparison of research interest variables by participation in Independent Study is shown in Table 4.

**Table 4:** Research Inclination by Independent Study participation.

**Variable**	**Undertaking Independent Study** ***n* (%)**	**NOT undertaking Independent Study** ***n* (%)**	***p-*value**
Age (years)	Mean: 30.15	Mean: 28.9	0.527
**Gender**			
Female	11 (77%)	15 (48%)	0.058
Male	3 (21%)	18 (52%)	
**Student status**			
Domestic	8 (57%)	9 (29%)	0.072
International	6 (43%)	22 (71%)	
**Current semester of study**			
First and second	2 (15%)	8 (58%)	*0.009
Third and fourth	11 (85%)	13 (42%)	
**Completed other research subjects**			
Yes	8 (67%)	8 (26%)	*0.032
No	4 (33%)	23 (74%)	
**Research completed under another degree (not MPH)**			
Yes	8 (57%)	15 (48%)	0.586
No	6 (43%)	16 (52%)	
**Research prior to UOW**			
Yes	6 (43%)	16 (52%)	0.586
No	8 (57%)	15 (48%)	
**Mentor**			
Yes	8 (57%)	4 (13%)	*0.004
No/Unsure	6 (43%)	27 (87%)	
**Research career**			
Yes	6 (43%)	11 (36%)	0.637
No/Unsure	8 (57%)	20 (65%)	
**Participate in research upon graduation**			
Yes	11 (79%)	23 (74%)	1
No/Unsure	3 (21%)	8 (26%)	
**Interest in projects outside MPH**			
Yes	11 (79%)	24 (77%)	1
No/Unsure	3 (21%)	7 (23%)	
**Confidence in doing research**			
Confident/Very Confident	5 (36%)	15 (48%)	0.428
Not Confident/Little Confident/Not Sure	9 (64%)	16 (52%)	
**Confidence in interpreting articles**			
Confident/Very Confident	9 (64%)	13 (42%)	0.165
Not Confident/Little Confident/Not Sure	5 (36%)	18 (58%)	

## Discussion

The results of this study suggest MPH students see improving the lives of the community as the most important purpose of research. Despite widespread interest in research among participants (76%), less than half (29%) of MPH students expressed interest in pursuing a career in research. The only variables associated with undertaking an Independent Study were the support of a mentor and previous enrolment in research subjects.

The findings of this study are in contrast with the research of medical students who cite individualistic motivations for conducting research such as career advancement and publishing research (2–8). A study of physicians undertaking an MPH in South Africa found the primary motivation was career progression (8). Altruistic motivations such as community health and well-being were not motivating factors identified in the research on medical students. This suggests that MPH students are driven by different motivations and could explain the comparatively high interest in research expressed by MPH students in Zweigenthal et al. and the present study in comparison to medical students (4, 8).

Low confidence in interpreting and doing research may indicate that a lack of perceived research skills is preventing students from pursuing a career in research. Several studies on medical students suggest that a lack of self-efficacy is a barrier to research (3, 5, 16). However, this study did not find evidence of a significant difference in research confidence between students undertaking an Independent Study compared to those not undertaking an Independent Study as part of their degree. This, combined with the discrepancy between interest in research and interest in a research career, suggests that other factors not measured in this study may be influencing student's aversion to pursuing a career in research.

The barriers to research in the literature examining medical students are more consistent with our findings. A lack of mentors has been identified as a barrier to participation in research among medical students (2–6, 9–15, 24). In the present study, students with the support of a mentor were more likely to be undertaking an Independent Study as part their degree than those without a mentor. Students with a mentor were also more likely to list improving the lives of the community as the most important purpose of research, suggesting a positive impact of mentor support on student's research motivations. Connecting students with mentors may also have positive impacts on students' motivations and inclination toward research.

This study is also consistent with research conducted with medical students suggesting that a lack of research training was a barrier to pursuing a career in research (2, 6, 9, 10, 12). Overall confidence interpreting and conducting research was low among this cohort of MPH students with less than half (44%) of students expressing confidence in doing research. Changes in the curricula may be required to improve students' research skills and confidence.

This study was a cross-sectional design limiting interpretation of the results. A longitudinal design may be able to examine if motivations and perceptions are intrinsic to students or can be influenced by changes in the curricula. The study may be subject to social desirability bias as it relied on the self-reported perceptions of students. With online anonymous surveys it is impossible to ensure that there is non-bias. There will always be some degree of bias in any study. We tried to minimize bias by having an independent person who was the Academic Program Director for the Masters of Public Health and not involved in this study write to the students and give them the link to the survey. This assured a degree of non-bias as someone not involved in the study invited them to participate and assured the students' responses were confidential etc. The anonymity of such surveys also means the students would be free to describe their true thoughts and feelings toward research and that the researchers would not be able to influence them unduly.

The research represents the perceptions of MPH students at one University and may not be representative of those from other locations. The response rate of 29% was low and resulted in a low sample size. The response rate is not unexpected as responses to web surveys can be on average 11% lower than other survey types (24). The low response rate may also be indicative of the cohort or part of a trend toward lower response rates due to response fatigue, among other factors (25–28). Despite the low response rate, a fair assumption is that those with an interest in research would participate in this study thereby over estimating the positive experiences and motivations toward research. These findings are still useful, as they would suggest that it is even more important that future curricula include interventions to support and encourage those who are interested in pursuing a research career.

## Conclusion

This study suggests MPH students see improving the lives of the community as the most important purpose of research. Despite widespread interest in research among participants less than half of MPH students expressed interest in pursuing a career in research. This contrasts with the research of medical students where students were motivated by career ambitions. Future research should explore why students express research interest but not interest in a research career. Changes in curricula and improved mentor support should be explored to increase interest in a research career.

## Data Availability Statement

The raw data supporting the conclusions of this article will be made available by the authors, without undue reservation.

## Ethics Statement

The studies involving human participants were reviewed and approved by The University of Wollongong Human Research and Ethics Committee. The patients/participants provided their written informed consent to participate in this study.

## Author Contributions

BC helped to draft the paper. CE and AA helped design the survey. JT analyzed the data. TH designed the research question and oversaw the entire project. All authors contributed to the article and approved the submitted version.

## Conflict of Interest

The authors declare that the research was conducted in the absence of any commercial or financial relationships that could be construed as a potential conflict of interest.

## Publisher's Note

All claims expressed in this article are solely those of the authors and do not necessarily represent those of their affiliated organizations, or those of the publisher, the editors and the reviewers. Any product that may be evaluated in this article, or claim that may be made by its manufacturer, is not guaranteed or endorsed by the publisher.
